# MIF homolog d-dopachrome tautomerase (D-DT/MIF-2) does not inhibit accumulation and toxicity of misfolded SOD1

**DOI:** 10.1038/s41598-022-13744-7

**Published:** 2022-06-10

**Authors:** Amina Alaskarov, Shir Barel, Shamchal Bakavayev, Joy Kahn, Adrian Israelson

**Affiliations:** Department of Physiology and Cell Biology, Faculty of Health Sciences and The Zlotowski Center for Neuroscience, Ben-Gurion University of the Negev, P.O.B. 653, 84105 Beer Sheva, Israel

**Keywords:** Neuroscience, Diseases, Molecular medicine

## Abstract

Amyotrophic lateral sclerosis (ALS) is a fatal neurodegenerative disease characterized by loss of upper and lower motor neurons. About 20% of familial ALS cases are caused by dominant mutations in SOD1. It has been suggested that toxicity of mutant SOD1 results from its misfolding, however, it is unclear why misfolded SOD1 accumulates within specific tissues. We have demonstrated that macrophage migration inhibitory factor (MIF), a multifunctional protein with cytokine/chemokine and chaperone-like activity, inhibits the accumulation and aggregation of misfolded SOD1. Although MIF homolog, D-dopachrome tautomerase (D-DT/MIF-2), shares structural and genetic similarities with MIF, its biological function is not well understood. In the current study, we investigated, for the first time, the mechanism of action of D-DT in a model of ALS. We show that D-DT inhibits mutant SOD1 amyloid aggregation in vitro, promoting the formation of amorphous aggregates. Moreover, we report that D-DT interacts with mutant SOD1, but does not inhibit misfolded mutant SOD1 accumulation and toxicity in neuronal cells. Finally, we show that D-DT is expressed mainly in liver and kidney, with extremely low expression in brain and spinal cord of adult mice. Our findings contribute to better understanding of D-DT versus MIF function in the context of ALS.

## Introduction

Amyotrophic lateral sclerosis (ALS) is a progressive neurodegenerative disease characterized by the loss of upper and lower motor neurons (MNs)^1^. ALS is the most well-known adult-onset motor neuron disease, with onset usually between 40 to 60 years of age. Symptoms of ALS include constantly progressive muscle atrophy and weakness, affecting respiratory muscles and thus reducing survival of most patients to 2– 4 years following disease onset^2^. Currently, there is no cure for ALS and only two medications are approved by the Food and Drugs Administration (FDA)—Riluzole (Rilutek)^3^ and Edaravone (Radicava)^4^, which provide only modest benefits in some early-stage patients.

Most ALS cases (about 90%) are sporadic (sALS)^5^ and occur in individuals with no prior family history. The remaining 10% of patients have at least one other affected family member and those cases are defined as familial ALS (fALS)^6^, which is symptomatically almost indistinguishable from sALS. There are several pathological mechanisms proposed to be involved in ALS, including nuclear disruptions^7^, protein loss of function^8^ or gain of toxic properties^9^, protein misfolding and aggregation^10^.

Zn/Cu superoxide dismutase (SOD1) is the first and the most-studied protein to be correlated with ALS^11^, associated with approximately 20% of fALS and 2% of sALS cases^12^. SOD1 is a 32 kDa homodimer, one of three superoxide dismutase enzymes found in humans^13^. Although conformational and functional changes of SOD1 have been proposed to induce motor neuron degeneration through several cellular mechanisms^14^, it has been suggested that SOD1 misfolding and/or aggregation is the most likely source of SOD1 toxicity in ALS^15^. Misfolded SOD1 can interact with various cellular proteins and interfere with their normal functions^16–19^. For instance, we have shown that misfolded SOD1 associates with the outer mitochondrial membrane, where it specifically binds to the voltage dependent anion channel (VDAC) through the N-terminal domain, affecting its conductance and compromising the energy supply of the cells^17,20^. Moreover, misfolded SOD1 was shown to affect the import of mitochondrial proteins^21^, affecting the normal function of the mitochondria. In the ER membranes, misfolded SOD1 was shown to interact with Derlin1 inducing ER stress^22^.

Several studies show the accumulation of the misfolded wild type SOD1 (SOD1^WT^) in sALS cases as well^23–29^. It appears that the minor changes in SOD1 methylation and post-transcriptional modifications might cause the wild type form of the protein to gain toxic properties and undergo misfolding^24,30,31^. At the same time, other studies reported the opposite findings, suggesting that misfolded SOD1 is not found in spinal cords^32–35^, lymphoblasts^36^, or CSF^37^ from sALS patients. Whatever the cause of ALS pathogenesis, whether due to a mutant or wild type SOD1 protein, the outcome is the accumulation of misfolded SOD1 and possibly its amyloid aggregation tendency, which is highly toxic to motor neurons.

Recently, in an attempt to determine the vulnerability of specific tissues toward misfolded SOD1 toxicity, our group identified the multifunctional protein macrophage migration inhibitory factor (MIF)^38^, whose levels were found to be extremely low within the spinal motor neurons^39,40^, and is thus suggested to be one of the causes for mutant SOD1 toxicity specifically in this neuronal population. MIF acts as a protein-folding chaperone in SOD1-fALS by significantly suppressing accumulation and amyloid aggregation of misfolded SOD1^39–41^. Moreover, MIF reduced mutant SOD1 association with the intracellular membranes of mitochondria and ER and extended survival of mutant SOD1-expressing motor neurons^39–41^. Finally, we have recently shown that overexpressing MIF in the spinal cord, by using AAV viral vectors, delayed disease onset and extended survival of two different mutant SOD1 mouse models of ALS^42^. In other studies, the MIF-SOD1 interaction was shown to be crucial not only in the context of ALS but also in cancer therapy. MIF was shown to be upregulated in different tumors^43^ and represents a biomarker and therapeutic target in multiple myeloma^44^. Specifically, MIF was shown to act as a chaperone for SOD1, thus suppressing misfolded SOD1 accumulation and maintaining SOD1 activity. This way, the cancer cells, which express high levels of MIF, become resistant to proteasome inhibitor treatment in human myeloma^44^.

In the current study we investigated D-dopachrome tautomerase (D-DT, also known as MIF-2), a newly discovered member of the MIF protein superfamily and MIF’s only known homolog^45^ , in the context of ALS. Although D-DT shares some structural and genetic similarities with MIF, its biological function is still not well understood, nor has it been studied in the context of ALS-related pathogenesis. D-DT is composed of 117 amino acids with a molecular weight of about 13 kDa^46–48^. Although, on the protein level D-DT and MIF share low amino acid sequence homology (34% identity in humans and 27% in mice), an X-ray crystal structure of D-DT has verified its 3D similarity with MIF, confirming that both proteins form a barrel-like structure homotrimer with 6 α-helices (2 per monomer) and 12 surrounding β-sheets (4 per monomer). Although both MIF and D-DT are functional tautomerases that catalyze the conversion of D-dopachrome to 5,6-dihydroxyindole-2-carboxylic acid (DHICA), D-DT also shows decarboxylase activity, converting it to 5,6-dihydroxyindole^49,50^. Among all MIF’s receptors, D-DT was shown to bind only to CD74, with even higher affinity than MIF, leading to the activation of ERK1/2 MAP kinase and downstream proinflammatory pathways^51^.

Although D-DT is gaining more attention in recent years, mostly regarding its cytokine pro-inflammatory activity in some autoimmune diseases and malignancies, not much is known yet about its role in neurological disorders. In the current study, we investigated a novel D-DT mechanism of action in SOD1-related fALS, while highlighting the similarities and differences between MIF and D-DT functions on mutant SOD1.

## Results

### Recombinant D-DT inhibits the formation of amyloid aggregates of mutant SOD1^G93A^

**Figure 1:**
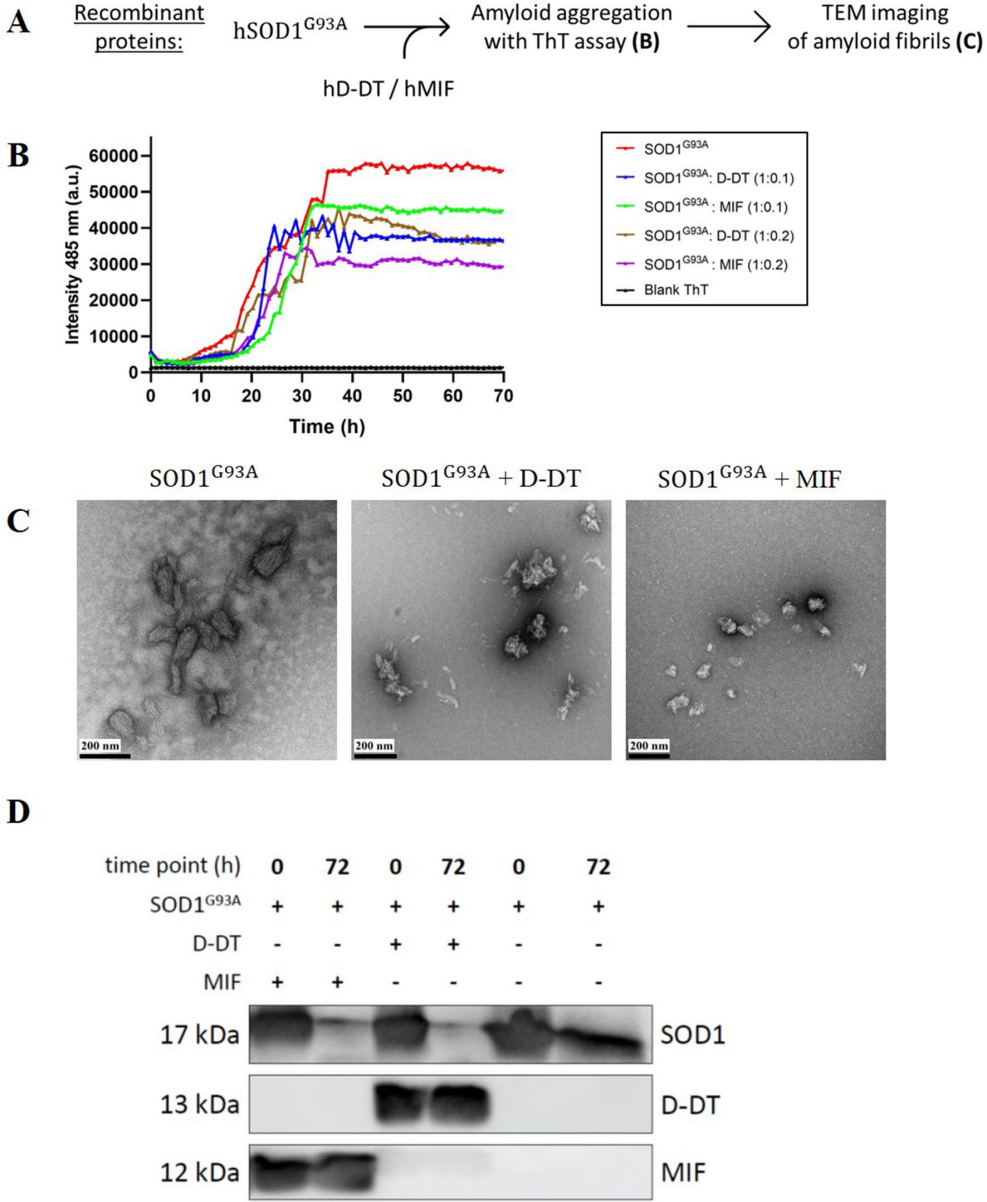
Recombinant D-DT inhibits the formation of mutant SOD1^G93A^ amyloid aggregates**.** (**A**) Schematic representation of the experimental protocol for determining whether recombinant D-DT, as compared to MIF, suppresses the amyloid aggregation of mutant SOD1. (**B**) ThT fluorescence was monitored for 70 h during the incubation of mutant SOD1^G93A^ (50 µM, red), either alone, or in the presence of recombinant D-DT (5 µM, blue; 10 µM, brown), or MIF^WT^ (5 µM, green; 10 µM purple). (**C**) TEM images of a SOD1^G93A^ (50 µM) incubated alone, or in the presence of recombinant D-DT (10 µM) or MIF^WT^ (10 µM). (**D**) Immunoblot showing the SOD1 monomer before and 72 h after incubation in aggregation inducing conditions alone or in the presence of recombinant human MIF or human D-DT.

Although the exact mechanism of MIF's protective activity on mutant SOD1 is not clear yet, we have previously shown that recombinant MIF^WT^, and its different variants (MIF^C60S^, MIF^P2A^ and MIF^N110C^) specifically suppressed the formation of mutant SOD1 (SOD1^G93A^ and SOD1^G85R^) amyloid fibrils and induced the formation of less toxic disordered amorphous aggregates^41^. Based on MIF and D-DT homologous quaternary homotrimer structure, we hypothesized that D-DT could have a similar effect on the formation of amyloid aggregates of mutant SOD1. The ALS model used in this study contains a mutation of glycine to alanine at amino acid position 93 of SOD1 (SOD1^G93A^). Transgenic mice expressing human SOD1^G93A^ develop a rapidly progressive motor neuron disease similar to fALS^52^, despite expression of endogenous mouse wild-type SOD1 (SOD1^WT^), with only a minor effect on the enzymatic activity of SOD1. To test whether D-DT affects mutant SOD1 amyloid formation, we purified mutant SOD1^G93A^ (Figure S1) and incubated it under aggregation-promoting conditions, with two different concentrations of recombinant human D-DT or human MIF, acting as a positive control (Fig. 1A). We measured amyloid aggregate formation by adding thioflavin T (ThT) to the protein solution and monitoring its fluorescent signal at 485 nm, for 70 h. ThT assay measures changes in fluorescence intensity of ThT upon binding to β-sheet rich structures, such as amyloid aggregates of SOD1^53,54^. Incubating mutant SOD1^G93A^, in the absence of recombinant MIF or D-DT, resulted in an exponential increase in ThT fluorescence intensity starting after 10–15 h of incubation (Fig. 1B). In contrast, incubating mutant SOD1^G93A^ in the presence of either recombinant human D-DT, or human MIF, suppressed the increase in the ThT signal in a similar manner (Fig. 1B). Supporting these findings is transmission electron microscopy (TEM) imaging, performed at the end of the 70 h experiment, revealing that whereas mutant SOD1^G93A^ formed fibrous aggregates when incubated alone, incubation with recombinant D-DT or MIF, suppressed this effect and promoted instead the formation of amorphous disordered aggregates (Fig. 1C). To validate these findings, we measured the consumption of SOD1 soluble protein after incubation of 72 h under aggregation-promoting conditions. The decrease of the SOD1 monomer band is similar with incubation of MIF and D-DT and it is even stronger than incubation of mutant SOD1 alone (Fig. 1D), suggesting that the total aggregation of SOD1 is not suppressed but only the amyloid one. Moreover, a turbidity assay to measure the total aggregation of SOD1 shows that the aggregation of mutant SOD1 in the presence of recombinant MIF or D-DT is not lower, but even higher than incubation of mutant SOD1 alone (Figure S2), strengthening our hypothesis regarding the formation of different type of aggregates. Interestingly, although recombinant MIF inhibits mutant SOD1 amyloid aggregation in a dose dependent manner, recombinant D-DT shows a similar inhibition at 5 µM or 10 µM (Fig. 1B). Incubating D-DT alone did not generate an increase in turbidity (Figure S2) or in the ThT signal, during 70 h of incubation (Figure S3).

### Recombinant D-DT does not reduce mutant SOD1^G93A^ misfolding

**Figure 2:**
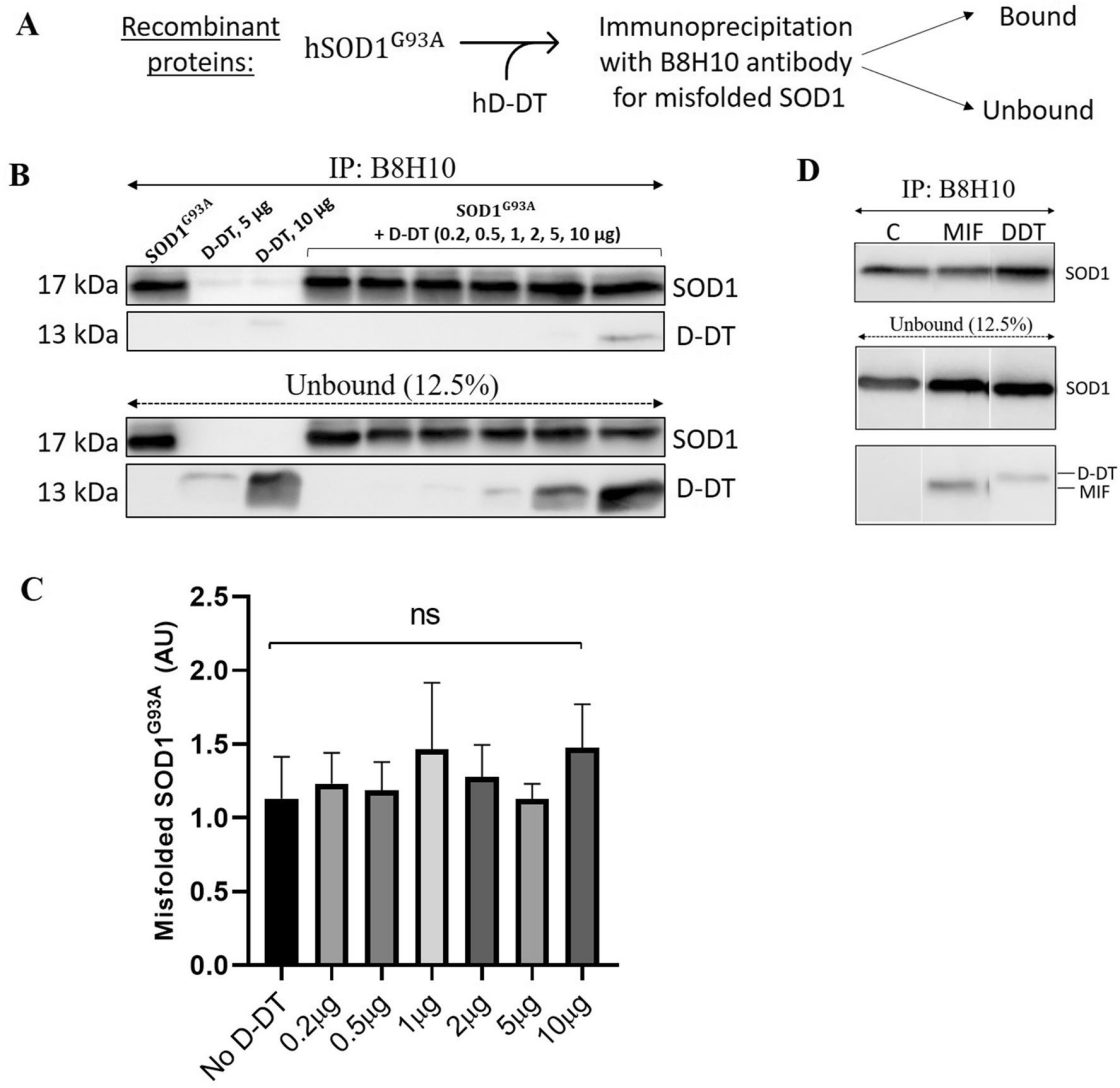
Recombinant D-DT does not affect mutant SOD1 misfolding. (**A**) Schematic representation of the experimental protocol using recombinant purified proteins. (**B**) The accumulation of misfolded SOD1 was determined by immunoprecipitation with B8H10 antibody, which recognizes misfolded SOD1, after incubating recombinant SOD1^G93A^ (2 µg) with increasing concentrations of recombinant D-DT (0.2, 0.5, 1, 2, 5, and 10 µg). Immunoblotting was used to determine the levels of SOD1 and D-DT present in the bound and unbound fraction of each immunoprecipitation assay. Data indicate one membrane out of 3 independent experiments. Full-length gels are presented in Supplementary Figure S4. (**C**) Quantification of immunoblotting bands with Evolution-Capt Edge software. Quantification analysis of the relative misfolded SOD1 (Bound/Unbound) performed with one-way ANOVA (n = 3). Bars represent mean ± SEM. (**D**) The accumulation of misfolded SOD1 was determined by immunoprecipitation with B8H10 antibody after incubating mutant SOD1 (2 µg) with recombinant MIF or D-DT (1 µg). Data indicate one representative experiment out of 3 independent experiments. The lanes from the right panel were not loaded in the same order as shown.

We have previously shown that MIF inhibits the accumulation of misfolded SOD1, both in vitro and in vivo^39,40,42^. Moreover, we demonstrated that MIF^N110C^, a previously reported cysteine mutant of MIF, which covalently locks MIF into its stable trimeric state, strongly co-precipitates with mutant SOD1, in contrast to MIF^WT^^41^. To investigate whether recombinant D-DT interacts with mutant SOD1 and reduces misfolded SOD1^G93A^ accumulation, similarly to MIF, we performed immunoprecipitation with B8H10 antibody, which recognizes various forms of misfolded SOD1. We incubated purified mutant SOD1^G93A^ alone, or in the presence of recombinant human D-DT in a dose-dependent manner (Fig. 2A). Moreover, we incubated recombinant D-DT alone, serving as a control for the specificity of B8H10 binding to the misfolded SOD1. Although D-DT co-precipitated with mutant SOD1^G93A^, as seen in the bound fraction for the higher concentrations only, using anti-D-DT antibody, it did not inhibit misfolded SOD1 accumulation, as recognized by immunoprecipitation with B8H10 antibody (Fig. 2B). Quantification analysis showed a non-significant difference in all the concentrations tested (Fig. 2C). In contrast to D-DT, recombinant MIF (1 µg) reduced mutant SOD1 misfolding at a lower dose (Fig. 2D).

### Overexpression of D-DT does not rescue mutant SOD1 toxicity in neuronal cultures

**Figure 3:**
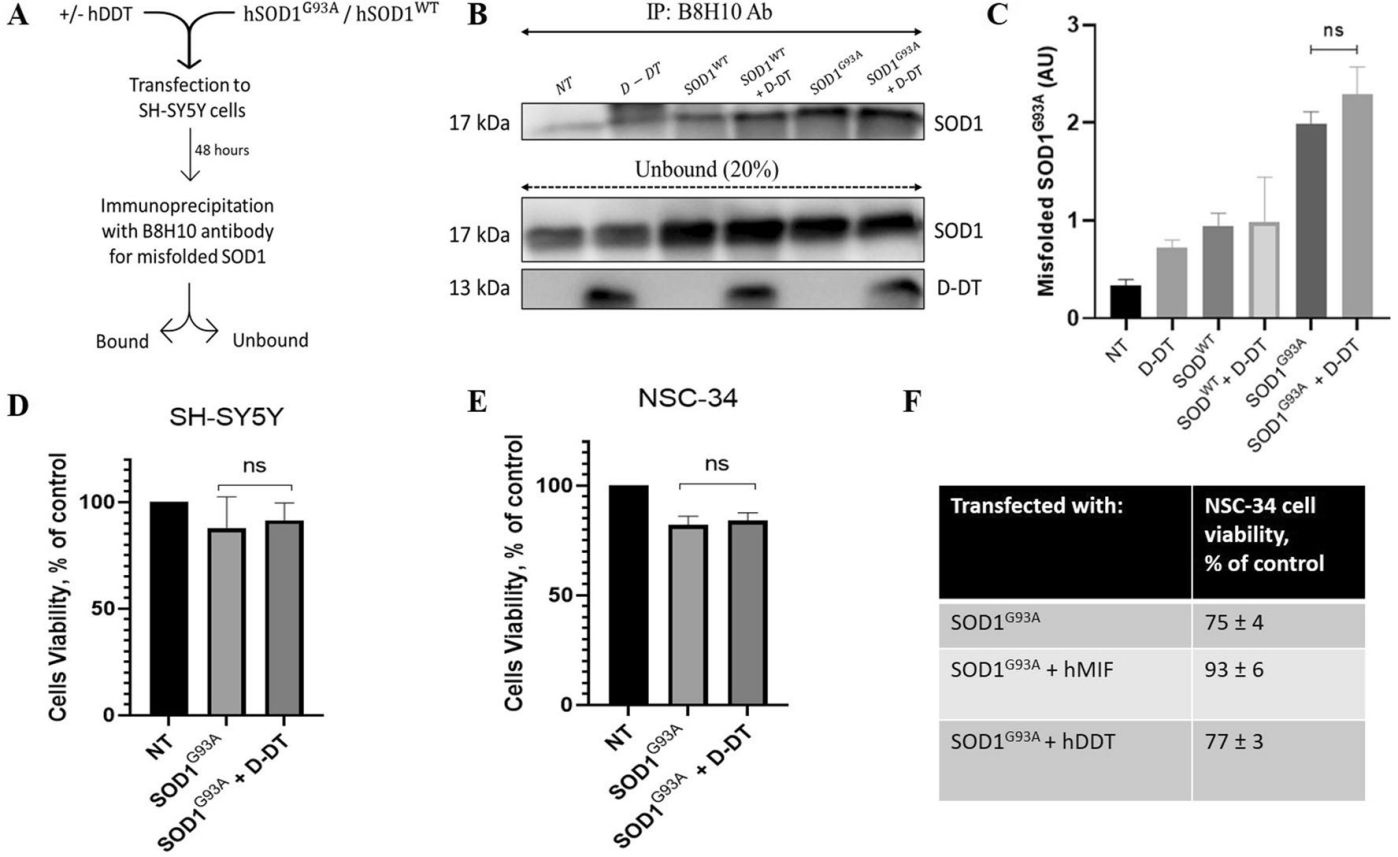
Overexpression of D-DT does not suppress the accumulation and toxicity of misfolded SOD1 in neuronal cells. (**A**) Schematic representation of the experimental protocol. D-DT-dependent inhibition of misfolded SOD1 accumulation was tested in SH-SY5Y cells, which were transfected to express SOD1^WT^ or mutant SOD1^G93A^, with or without D-DT. (**B**) Misfolded SOD1 was detected by immunoprecipitation with the B8H10 antibody. Immunoblotting was used to determine the levels of SOD1 and D-DT present in the bound and unbound fraction of each immunoprecipitation assay. NT represent non-transfected cells. Data indicate one membrane out of 3 independent experiments. Full-length gels are presented in Supplementary Figure S5. (**C**) Quantification of immunoblotting bands with Evolution-Capt Edge software. Quantification analysis of the relative misfolded SOD1 (Bound/Unbound) performed with one-way ANOVA (n = 3). Bars represent mean ± SEM. A cell-survival analysis was performed with ELISA at 490 nm in (**D**) SH-SY5Y and (**E**) NSC-34 neuronal cells, 48 h after transfection with mutant SOD1^G93A^ alone, or in the presence of human D-DT. NT represent non-transfected cells. Quantitative analysis from triplicates of different biological experiments was performed with one-way ANOVA (n = 3). Bars represent mean ± SEM. (**F**) Table comparing the effects of D-DT and MIF on cell viability of NSC-34 cells.

Expressing mutant SOD1^G93A^ in neuronal cells reduces cellular survival by approximately 25%^39–41^. We have previously reported that this toxic effect is reversed by co-expressing MIF^WT^ or any of the three MIF variants tested in SH-SY5Y or motor neuron-like NSC-34 cells^39–41^. Moreover, MIF inhibited mutant SOD1 misfolded accumulation as detected by different anti-misfolded SOD1 antibodies^39–41^. To determine whether D-DT suppresses the accumulation of misfolded SOD1 and protects mutant SOD1-induced toxicity in neuronal cells, we overexpressed human D-DT in SH-SY5Y cells. As a control, we co-transfected D-DT in the presence or absence of SOD^WT^. Forty-eight hours post transfection, we performed immunoprecipitation with a B8H10 antibody for misfolded SOD1 (Fig. 3A). We report that overexpressing D-DT in neuronal cells did not reduce misfolded SOD1 accumulation as detected with the B8H10 antibody (Fig. 3B-C). Moreover, overexpressing mutant SOD1^G93A^ in SH-SY5Y or in motor neuron-like NSC-34 neuronal cells decreased cellular survival by approximately 20% without any significant rescue in the presence of D-DT (Fig. 3D-E). In contrast to its homolog MIF, D-DT does not inhibit neither misfolded SOD1 accumulation nor mutant SOD1 cellular toxicity (Fig. 3F).

### Overexpressing D-DT in neuronal cells does not inhibit aggregation of mutant SOD1

**Figure 4:**
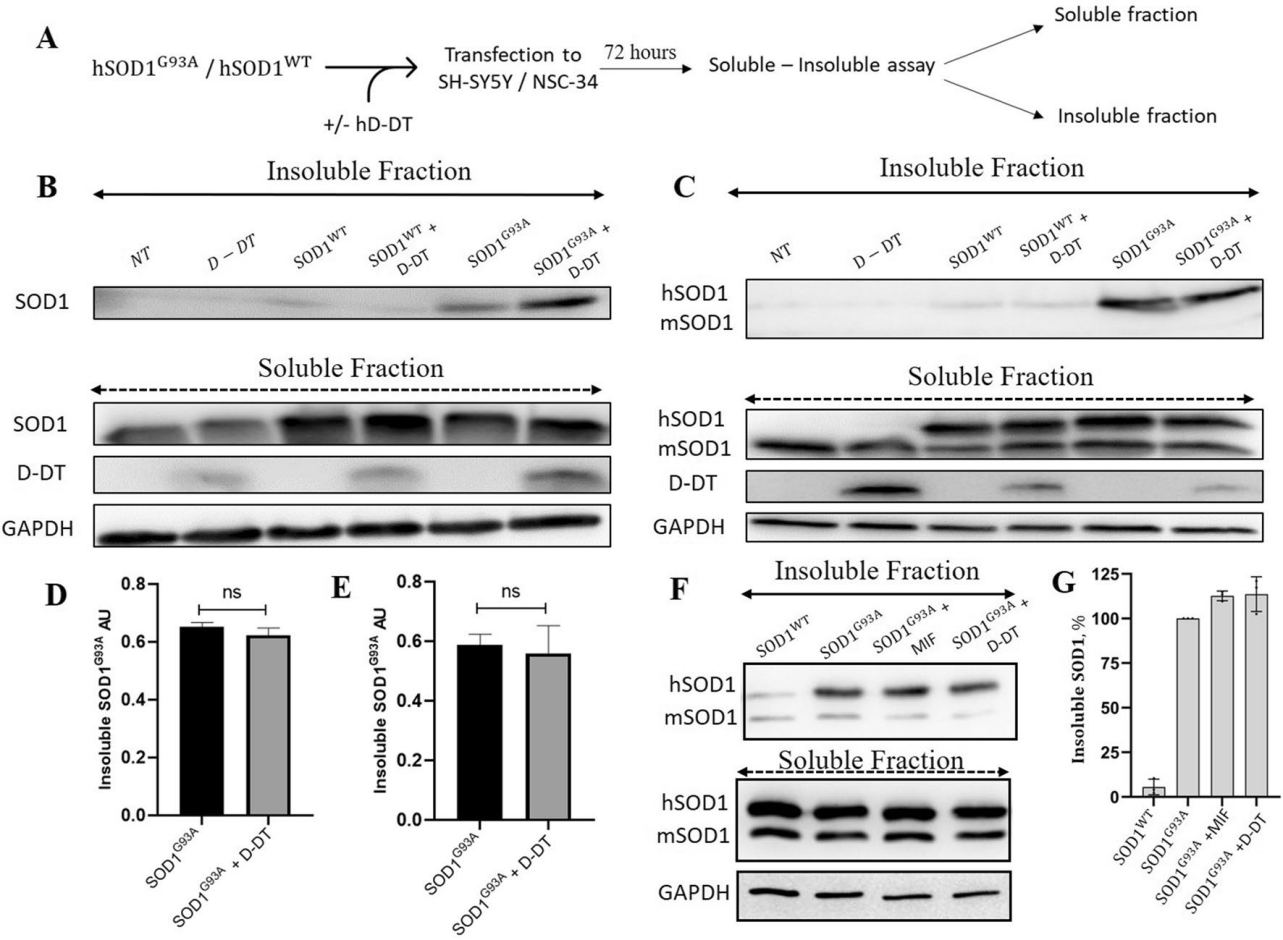
Overexpression of D-DT does not affect mutant SOD1 aggregation in SH-SY5Y and NSC-34 cells. (**A**) A schematic representation of the experimental protocol. (**B, C**) Immunoblots of the soluble and insoluble fractions of SH-SY5Y (**B**) and NSC-34 (**C**) cells transfected for 72 h with vectors for SOD1^WT^, SOD1^G93A^, D-DT or co-transfected with 2 vectors (SOD1^WT^ + D-DT and SOD1^G93A^ + D-DT). NT represent non-transfected cells. Anti-GAPDH antibody was used to detect total protein loading amounts. Note that in the NSC-34 cells immunoblot (**C**) mouse SOD1 (mSOD1) migrates faster than the overexpressed human SOD1 (hSOD1). Data indicate one membrane out of 3 independent experiments. Full-length gels are presented in Supplementary Figure S6. (**D, E**) Quantification of the band intensity representing the insoluble SOD1 in SH-SY5Y (**D**) and NSC-34 (**E**) cells performed with Evolution-Capt Edge software. Quantification analysis of the relative aggregation propensity performed with student t-test. Bars represent mean ± SEM. (**F**) Immunoblots of the soluble and insoluble fractions of NSC-34 cells transfected for 72 hours with vectors for SOD1^WT^, SOD1^G93A^ or co-transfected with 2 vectors (SOD1^G93A^ + MIF and SOD1^G93A^ + D-DT). Anti-GAPDH antibody was used to detect total protein loading amounts. (**G**) Quantification of the band intensity representing the insoluble SOD1 in NSC-34 cells in F performed with Evolution-Capt Edge software. Quantification analysis of the relative aggregation propensity performed with student t-test. Bars represent mean ± SEM.

Noxious SOD1 forms insoluble protein aggregates which are highly toxic to the neuronal cells^55^. As we have shown above, recombinant D-DT inhibited the formation of mutant SOD1 amyloid fibrils while altering the morphology to amorphous aggregates (Fig. 1). To determine whether D-DT has the ability to suppress the aggregation of mutant SOD1 in neuronal cells, we overexpressed mutant SOD1^G93A^ in SH-SY5Y and NSC-34 cells, in the presence or absence of human D-DT overexpression. Seventy-two hours later, we separated cytosolic soluble proteins from the insoluble protein aggregates by performing a soluble-insoluble assay (Fig. 4A). Overexpression of human D-DT did not significantly decrease mutant SOD1 aggregation, as seen in the immunoblots of insoluble fractions, in both SH-SY5Y (Fig. 4B, D) and NSC-34 (Fig. 4C, E) neuronal cells. Similar results were observed by overexpressing MIF in mutant SOD1 expressing NSC-34 cells (Fig. 4F, G). As a control we co-transfected neuronal cells with SOD1^WT^, with or without D-DT, which did not form protein aggregates as detected in the insoluble fractions immunoblots (Fig. 4B, C, F, G).

### Endogenous D-DT levels are extremely low in the brain and spinal cord of adult mice

**Figure 5:**
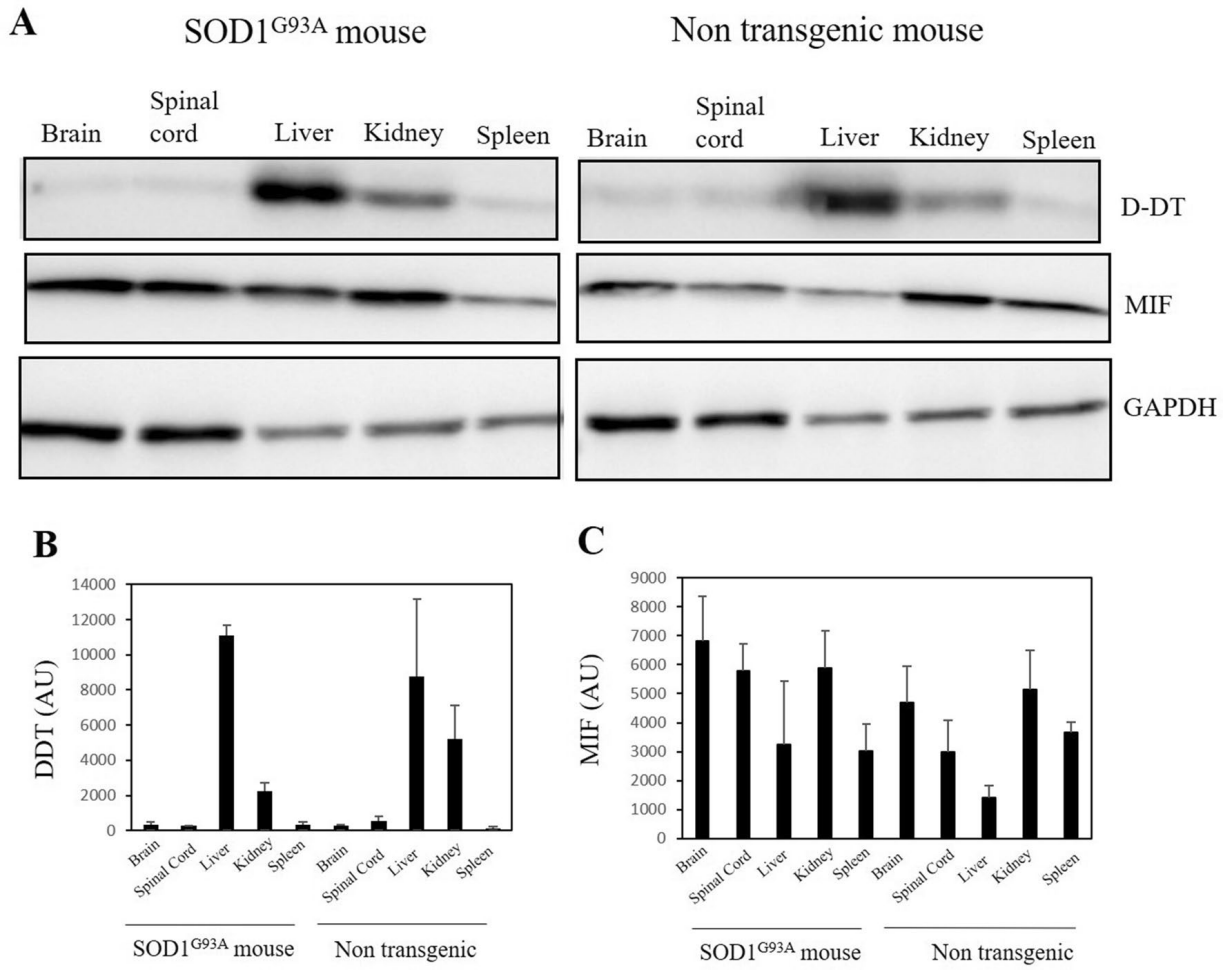
Endogenous D-DT level is extremely low in the brain and the spinal cord of the adult mice. (**A**) Endogenous D-DT expression is detected mainly in the liver and kidney of 4.5 months-old SOD1^G93A^ ALS mice and age-matched healthy mice. Endogenous MIF is expressed at higher levels in the brain, spinal cord and spleen and lower levels in the liver compared with D-DT. Anti-GAPDH antibody was used to detect total protein amounts. (**B, C**) Quantification of the expression level of endogenous D-DT (**B**) and MIF (**C**) from 3 different mice performed with Evolution-Capt Edge software. Bars represent mean ± SEM. Full-length gels are presented in Supplementary Figure S7.

We have previously shown that although MIF is expressed in the brain and the spinal cord, motor neurons accumulate extremely low levels of MIF^39,40^. Endogenous D-DT expression was previously shown in mouse tissue organs^51,56,57^, with the highest expression in the liver and testis. We investigated and compared D-DT and MIF expression in different tissues of the SOD1^G93A^ transgenic and age-matched (4.5 months) healthy mice. Endogenous D-DT expression was detected mainly in the liver and kidney tissues, in both the mutant SOD1^G93A^ and healthy mice (Fig. 5A, B). We could almost not detect endogenous D-DT expression in the brain, spinal cord, or spleen tissues of both mice. In contrast, endogenous MIF was detected in all tissues at relatively high levels (Fig. 5A, C).

## Discussion

In the current study, we investigated D-dopachrome tautomerase (D-DT or MIF-2), a second member of the MIF protein superfamily and known homolog to MIF, encoded by the *DDT* gene^45^, in the context of ALS pathogenesis. In mammals, D-DT is involved in numerous pathological roles in inflammatory, autoimmune, and chronic respiratory diseases^57–59^. In addition, several studies investigated pathogenetic roles of MIF, and possibly D-DT, in different types of cancer, including neuro- and glioblastoma^60–62^. This is the first study that demonstrates a D-DT mechanism of action in neurodegenerative diseases, specifically in SOD1-related fALS. We investigated whether similarly to MIF, D-DT protects neuronal cells from toxic misfolded SOD1 accumulation and aggregation, acting as a protein-folding chaperone on mutant SOD1. Specifically, as observed for MIF, we demonstrate that recombinant D-DT inhibits misfolded SOD1 amyloid aggregation by changing the pattern from amyloid to amorphous aggregates. However, in contrast to MIF, overexpression of human D-DT in neuronal cultures neither reduces misfolded SOD1 accumulation and aggregation nor protects these cells from mutant SOD1 induced toxicity. Finally, we show that endogenous D-DT, in contrast to MIF, is expressed at extremely low levels in the spinal cord and brain of adult mice.

As mentioned previously, mutant and wild type SOD1 might misfold and aggregate into amyloid fibrils under certain conditions^63–65^. Several studies have demonstrated the general toxicity of amyloid aggregates^66,67^. We show that recombinant D-DT inhibits the amyloid aggregation of mutant SOD1^G93A^, as observed by the ThT assay, and instead changed the aggregation pattern from fibril-like aggregates to amorphous disordered ones, as detected by TEM and validated by turbidity assay and decrease of the soluble SOD1 monomer. The ability of D-DT to transform the aggregation pattern of mutant SOD1 to non-fibril, disordered aggregates is an important finding since it was shown that these forms of aggregates are less toxic to the cells than amyloid aggregates^68^. In a cellular context, overexpression of D-DT in mutant SOD1^G93A^ overexpressing neuronal cells (SH-SY5Y and NSC-34), did not affect mutant SOD1 aggregation, as recognized by a soluble–insoluble assay. This is not surprising since both MIF and D-DT do not suppress the total amount of aggregates, but change their pattern. Moreover, we showed that D-DT, when incubated alone, does not form aggregates with itself, as tested by ThT assay and in neuronal cells. Hence, we suggest that this inhibitory effect of D-DT on mutant SOD1 amyloid aggregation (seen only with recombinant proteins) is caused by a possible unstable direct interaction between D-DT and mutant SOD1, which is probably affected by other, currently unknown, intracellular factors.

**Figure 6:**
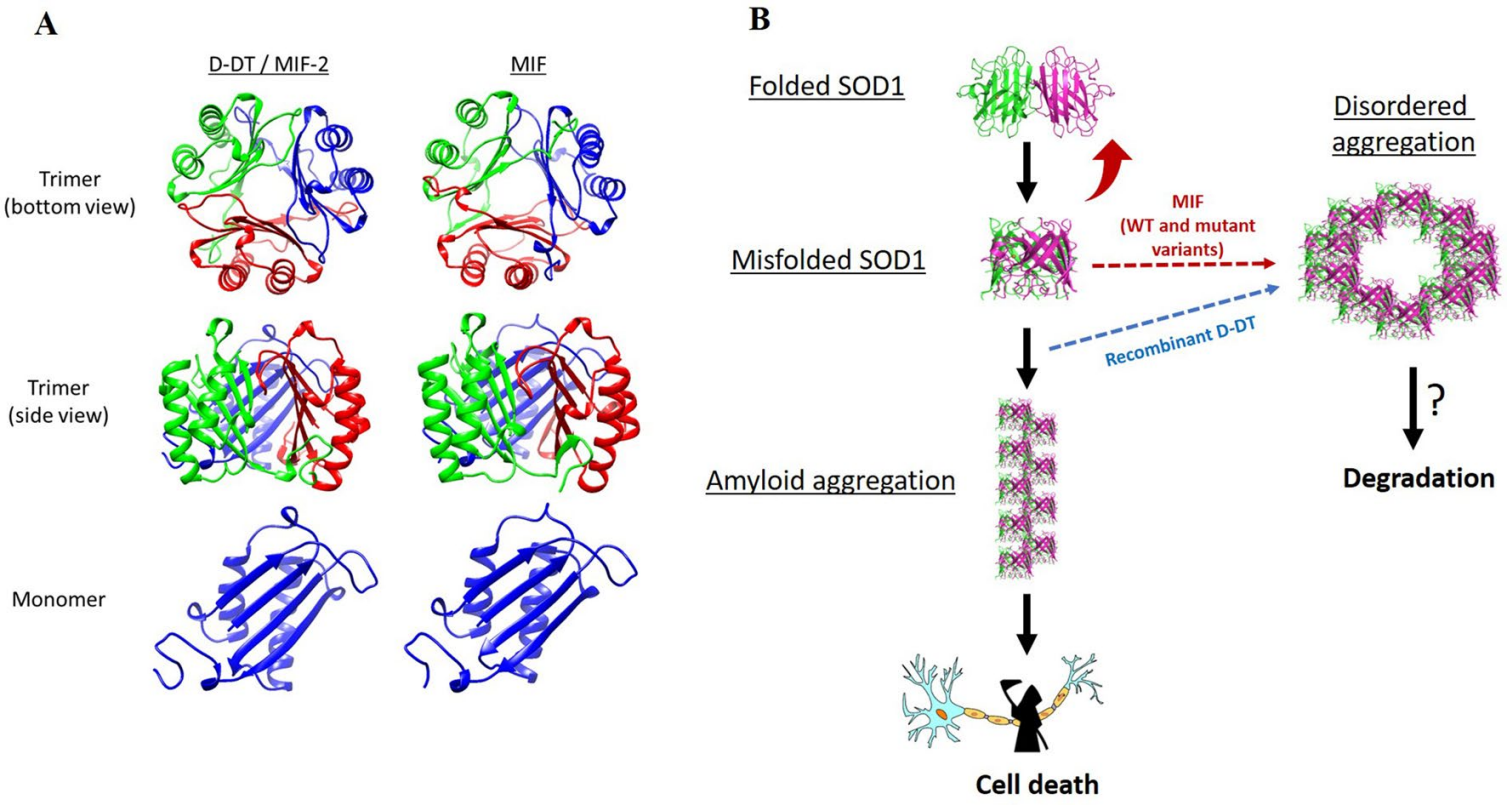
D-DT versus MIF, proposed mechanism in SOD1-related fALS. (**A**) 3D structures of human D-DT/MIF-2 (left panel) and human MIF (right panel), presented as trimer (top panel—bottom view, middle panel—side view) and monomer (bottom panel). These figures were prepared with UCSF Chimera and PDBePISA, based on MIF and D-DT crystal structures available in RCSB Protein Data Bank (PDB ID: 1MIF and 1DPT). (**B**) Mutant or wild type SOD1 under stress conditions accumulates as a toxic misfolded protein, which may aggregate by forming toxic amyloid fibrils. By interacting with mutant SOD1, MIF prevents its misfolding and cellular toxicity. In contrast, recombinant D-DT interacts with misfolded SOD1 at a later phase, thus it is not able to reduce misfolded SOD1 accumulation and toxicity, but inhibits mutant SOD1 amyloid aggregation promoting instead the formation of disordered aggregates.

N110C is a cysteine mutant of MIF (MIF^N110C^), which covalently locks MIF into its trimeric state while losing its CD74-dependent cellular signaling ability as a cytokine^69,70^. Previously, our group demonstrated that MIF^N110C^ strongly suppresses misfolded SOD1 accumulation. Moreover, we and others have shown that MIF^N110C^, in contrast to MIF^WT^, has a stronger affinity for mutant SOD1, as observed by SPR analysis or immunoprecipitation assay^41,44^, suggesting that the MIF chaperone-like effect is more active with the trimeric form of the protein. In the current study, we show that recombinant D-DT, co-precipitates with mutant SOD1^G93A^ only at very high concentrations, as observed by immunoprecipitation with a specific conformational antibody for misfolded SOD1, suggesting that D-DT and MIF have a similar affinity for mutant SOD1. However, in contrast to MIF, recombinant D-DT neither reduces misfolded SOD1 accumulation, as revealed by immunoprecipitation with recombinant proteins and in neuronal cells, nor protects mutant SOD1 neuronal cells from misfolded SOD1 toxicity. We suggest that D-DT affects mutant SOD1 aggregation only at a later stage when SOD1 is already transformed to an oligomeric, probably misfolded form. In contrast, MIF likely binds to soluble, non-aggregated SOD1, preventing its early misfolding accumulation. This difference in the interaction patterns between MIF-SOD1 and D-DT-SOD1 may lie in the small differences in their protein structures. One example is the region surrounding the active pocket of the two proteins. While MIF is positively charged in the surrounding area of the active site, D-DT is negatively charged in the same region^71^. Moreover, D-DT lacks the ELR (Glu-Leu-Arg) motif, which is present in MIF, and is essential for most receptor-ligand interactions^72^ (Fig. 6A). Another difference lies in the potential metal binding sites. In D-DT only one potential metal ligand residue is present (D6), as compared to two metal ligand residues in MIF (N7 and Q46). The one missing in D-DT is on the entrance to the central channel.

Finally, we investigated endogenous D-DT expression in adult mutant SOD1 and age-matched healthy mice. Previously, we have shown that spinal motor neurons, which are mostly affected in ALS, accumulate extremely low levels of MIF, which is believed to be one of the reasons for mutant SOD1 toxicity in these cells^39^. Here, we show that endogenous D-DT is expressed strongly in the liver and kidney of the adult mice, in both mutant SOD1 ALS model and healthy mice. However, in contrast to MIF, we have detected extremely low endogenous D-DT expression in central nervous system tissues including the brain and spinal cord. Interestingly, we have not found any endogenous D-DT expression in the non-transfected neuronal cell-lines as well, including SH-SY5Y and motor neuron-like NSC-34 cells. According to previous findings, mRNA levels of D-DT seem to decrease in the adult mice in all organs except the kidney^56^. Changes in mRNA expression might be correlated with changes in protein levels, suggesting that the half-life of the D-DT protein may be short in most adult tissues.

During the last decades, D-DT and MIF mechanisms have been investigated in multiple human pathologies. While most of the studies have been directed to cancer or autoimmune diseases, we believe that these “cytokine-twins” should be considered also in neurodegenerative diseases, such as ALS. In our study, we propose a novel mechanism for D-DT activity in SOD1 related fALS, as compared to previously established MIF chaperone-like activity on mutant SOD1 (Fig. 6B). Although this is only a speculation at this stage, we suggest that MIF, in contrast to D-DT, acts as a chaperone for the soluble form of misfolded SOD1, preventing its accumulation and further association with intracellular membranes and cellular toxicity. In contrast, D-DT likely interacts with misfolded SOD1 species at a later stage, being unable to inhibit the accumulation of misfolded SOD1 and its toxicity. The elucidation of the exact mechanism by which recombinant D-DT interacts with mutant SOD1 (*G93A* or possible other variants) and suppresses its amyloid aggregation, requires further investigation. Our findings might contribute to a better understanding of misfolded SOD1 pathogenic mechanisms and the possible implications of the MIF superfamily chaperones as a therapeutic approach in SOD1-related ALS, and other pathologies where SOD1-MIF interactions play a major role.

## Materials and methods

All methods are reported in accordance with ARRIVE guidelines in this manuscript.

### Recombinant SOD1^G93A^ purification

The human SOD1^G93A^ protein was produced in *E.coli* BL-21 cells, grown without Zn^2+^ and Cu^2+^ supplements and purified to homogeneity under non-denaturing conditions, as described previously^73^ (Figure S1).

### Thioflavin-T (ThT) aggregation assay

Human recombinant SOD1^G93^^A^ protein (50 μM), with or without human D-DT (0.2–10 µM), or human MIF^WT^ as a control, were incubated in 200 μl condition mix [200 mM HEPES buffer × 4, 400 mM NaCl pH 7.4, 5 mM EDTA, 1 mM Tris(2-carboxyethyl)phosphine hydrochloride (TCEP, Sigma)] with 50 μM Thioflavin T (Sigma) in a black 96-well plate (flat bottom, black non-binding, Greiner) at 37 °C with fast continuous shaking using ELISA reader as described previously^41^. All samples were performed in triplicates and the fluorescence (excitation at 440 nm, emission at 485 nm) was measured at 15 min intervals for 72 h by Infinite M200 Pro (Tecan).

### Transmission electron microscopy (TEM) imaging

Samples for TEM imaging were prepared as described elsewhere^74^. Briefly, at the end of the ThT aggregation assay (72 h), 2.5 μl samples were deposited on a carbon-coated copper 300 grid. After 1 min, the excess liquid was carefully blotted onto filter paper, which was then dried at ambient temperature for 1 min. Uranyl acetate (5 μl, 2%) was added to the grid and, after 1 min, the excess of the salt solution was carefully removed with a filter paper. The imaging was performed by the Nano-Fabrication Center team at Ben-Gurion University using a ThermoFisher Scientific (FEI) Talos F200C transmission electron microscope with an acceleration voltage of 200 kV. The images were taken with Ceta 16 M CMOS camera, and different magnifications (100–500 nm) were used, depending on the size of the fibril aggregates. The visible features were sensitive to the electron beam exposure, indicating their organic origin.

### Plasmids

All SOD1 plasmids, including pCI-hSOD1^WT^ and pCI-hSOD1^G93^^A^ were generated previously by our group, by inserting human *SOD1* constructs into the pCI-NEO vector (Promega), between the EcoRI and the NotI sites. A *DDT* expressing plasmid pET-21b-hDDT, was generously received from the Bernhagen group (Munich, Germany). The sequence of human *DDT* was transferred from the pET-21b vector^75^ into the mammalian expression vector pcDNA3.1 (-) vector, between the BamHI and NotI restriction sites. Human *DDT* gene was amplified by PCR from pET-21b vector using following primers:

Forward primer: 5’-ATTACGCGGCCGCCACCATGCCGTTCCTGGAG-3’.

Reversed primer: 5’-GCTAGGATCCCTATAAAAAAGTCATGAC-3’.

### Cell culture and transfection

SH-SY5Y (human neuroblastoma cells) were received from the laboratory of Prof. Shoshan-Barmatz (Ben-Gurion University), HEK-293 T (human embryonic kidney cells) were purchased from the ATCC (American Type Culture Collection), and NSC-34 (mouse motor neuron-like cells) cells were received from the laboratory of Prof. Offen (Tel Aviv University). The cells were cultured at 37 °C and 5% CO_2_ in Dulbecco’s modified Eagle medium (DMEM) supplemented with 10% tetracycline-free fetal bovine serum (FBS), 1% L-Glutamine and 1% Penicillin–Streptomycin Solution (all reagents from Biological Industries). Transfection was performed using TurboFect™ transfection reagent (Thermo Fisher Scientific, Inc., Lithuania) according to the manufacturer’s protocol. Analysis was performed usually after 24, 48, and 72 h.

### Immunoblotting

Desired proteins (1–50 µg) were separated on a 15% SDS-PAGE gel in running buffer (25 mM Tris, 192 mM Glycine, 0.1% SDS) in a Bio-Rad mini-PROTEAN® Tetra system. Separated proteins were then transferred to nitrocellulose membrane using a cold transfer buffer (25 mM Tris, 192 mM Glycine, 20% methanol) in a Bio-Rad mini-PROTEAN® Tetra system (1 h at 200 mA). Membranes were stained with Ponceau-s (Sigma) to visually verify protein quantity. Following and then probed overnight with various primary antibodies, including sheep anti-DDT (1:625, R&D systems), rabbit anti-DDT (1:500, Nobusbio), mouse anti-SOD1 (1:250, 24, Santa Cruz), mouse anti-GAPDH (1:200, Santa Cruz) and rabbit anti-MIF (1:1000, Abcam). Horseradish peroxidase^72^—conjugated anti-sheep, anti-rabbit, or anti-mouse IgG secondary antibodies (Jackson Immunochemicals) were used and detection was done with the EZ-ECL Chemiluminescence Detection kit for HRP (Biological Industries, Kibbutz Beit-Haemek, Israel). The membranes were photographed by Fusion Solo X biomolecular imager (Ivilber). Reproving of the membranes was performed with a Restore™ PLUS Stripping Buffer (Thermo) for 15 min, followed by standard TBST and TBS (× 2) washes.

### Immunoprecipitation (IP)

Whole-cell extracts (100 µg), or purified proteins (0.2–10 µg), were solubilized in cold IP buffer (0.05 M Tris pH 7.4, 0.4 M NaCl, 0.5% Nonidet P-40) and incubated overnight at 4 °C, while rotating, with B8H10 antibody for misfolded SOD1 (MediMabs), previously crosslinked to Dynabeads™ protein G (Thermo) and diluted in PBS with 0.02% Tween-20 according to the manufacturer’s protocol. Following magnetic separation, unbound samples were boiled for 5 min and withdrawn for immunoblotting analysis. Bound samples were eluted by boiling in 2 × sample buffer for 5 min and withdrawn for immunoblotting analysis.

### Soluble – insoluble assay

72 h post transfection, cultured cells were placed on ice, medium removed and washed (× 2) with PBS × 1. Cells were detached with 1 ml of the cold Soluble Buffer (PBS × 1, 5 mM EDTA, 1% Triton × 100, 10% Glycerol, 1 mM PMSF, 1% PI) and incubated on ice for 20 min. Detached cells were collected into an Eppendorf tube and centrifuged at 17,000 × g for 30 min at 4 °C. Supernatant cytosolic fraction, containing soluble proteins, was collected, boiled for 5 min at 95 °C in sample loading buffer × 5 and withdrawn for immunoblotting analysis. Protein concentration of soluble protein fraction was measured by the Bradford (Bio-Rad) method using bovine serum albumin as standard.

The remaining pellet was resuspended in 1 ml of the cold Soluble Buffer and centrifuged at 17,000 × g for another 30 min at 4 °C. After the removal of the supernatant fraction, the pellet, containing insoluble proteins, was resolved in 100 µl of 8 M Urea and sonication (Elmasonic S30H, Elma) for 45 min. After the disappearance of the insoluble pellet, the fraction was boiled for 5 min at 95 °C in sample loading buffer × 5, and withdrawn for immunoblotting analysis. Protein concentration of insoluble protein fraction was measured by the BCA assay (Cayman Chemical).

### Animals

All experiments were carried out in accordance with the Israeli legislation for research on animals. All efforts were taken to minimize animal suffering. B6-TgN-SOD1-G93A-1Gur were obtained from Jackson Laboratory (Maine, US) and bred in our animal facility. For both SOD1 mutant and non-transgenic mice, the protocol was approved by the Animal Care and Use Committee of Ben-Gurion University of the Negev, as required by Israeli legislation.

### Tissue harvesting and protein extraction

Mice were anesthetized by inhalation of 1.5–3% isoflurane. Brain, spinal cord, spleen, kidney and liver tissues were dissected out and homogenized on ice in 3 volumes of ice-cold lysis buffer [150 mM NaCl, 20 mM Tris–HCl pH 7.5, 1 mM PMSF, 1% Triton × 100, 1% PI (APExBio), 0.5% Sodium Deoxycholate, 0.1% SDS]. The homogenates were centrifuged at 5000 × g for 30 min at 4 °C. Supernatant cytosolic fraction was collected, fast frozen in liquid nitrogen, and stored at − 80 °C until use. Protein concentration was measured by the Bradford (Bio-Rad) method using bovine serum albumin as standard.

### Cell viability assay (MTS)

Cell viability was performed using CellTiter 96® AQueous One Solution Cell Proliferation Assay (Promega) according to the manufacturer’s protocol. Briefly, 24 h after transfection, cells were trypsinized, resuspended in 3 ml fresh DMEM, medium, seeded in a 96-well plate (7,000 cells per well) and incubated at 37 °C for another 24 h in a humidified, 5% CO_2_ atmosphere. The following day, 20 µl of CellTiter 96® AQueous One Solution Reagent was added to each well. Absorbance at 490 nm was measured at 5 min intervals for 1 h by Infinite M200 Pro (Tecan). All samples were performed in triplicates.

### Statistical analysis

Quantification of band intensity across experiments was performed using Evolution-Capt Edge software. The data was transferred and statistically analyzed using GraphPad Prism (La Jolla, CA). Values are reported throughout as mean ± SEM. Student’s t-tests and one-way ANOVA were used to compare datasets, after confirming a normal distribution of the data by using the Shapiro–Wilk normality test. Significance was set at a confidence level of 0.05, while NS (non-significant) denotes p ≥ 0.05.

## Supplementary Information

Supplementary Information.
