# Supplementary material for: MIF homolog d-dopachrome tautomerase (D-DT/MIF-2) does not inhibit accumulation and toxicity of misfolded SOD1
Source: Sci Rep. 2022 Jun 10;12:9570. doi: 10.1038/s41598-022-13744-7 (PMC9187739; doi:10.1038/s41598-022-13744-7)
Supplement: Supplementary file 1 — Supplementary Information. [file 41598_2022_13744_MOESM1_ESM.pdf]

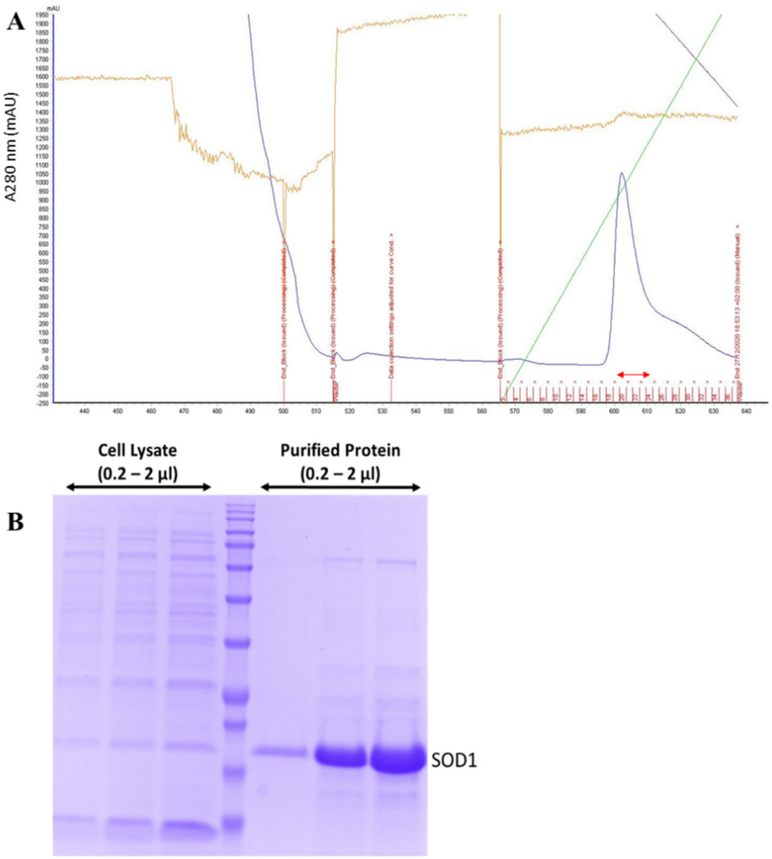

Figure S1: SOD1^{G93A} protein purification. (A) Purification graph using ÄKTA pure protein purification system. Cell lysate was subjected to a 5 ml HisTrap FF column and equilibrated with the binding buffer. The column was washed, and the protein was eluted with an elution buffer by an increasing imidazole gradient. Purest SOD1^{G93A} containing fractions (19-24, pointed out with a red arrow) were collected. **(B)** Coomassie staining of purified mutant SOD1 before and after the purification, SDS-PAGE gel (15%).

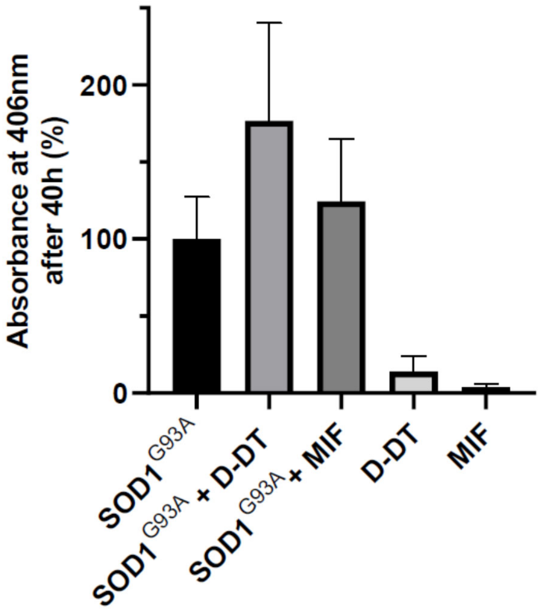

Figure S2: Turbidity measured at 406 nm indicates the formation of SOD1^{G93A} aggregates in solution during shake-incubation at 37 °C, in the absence or presence of 10 µM recombinant D-DT or recombinant MIF. The turbidity of D-DT and MIF alone is also shown as a control. Of note, in this D-DT and MIF concentration range, significant inhibition of ThT fluorescence elicited by mutant SOD1^{G93A} is seen.

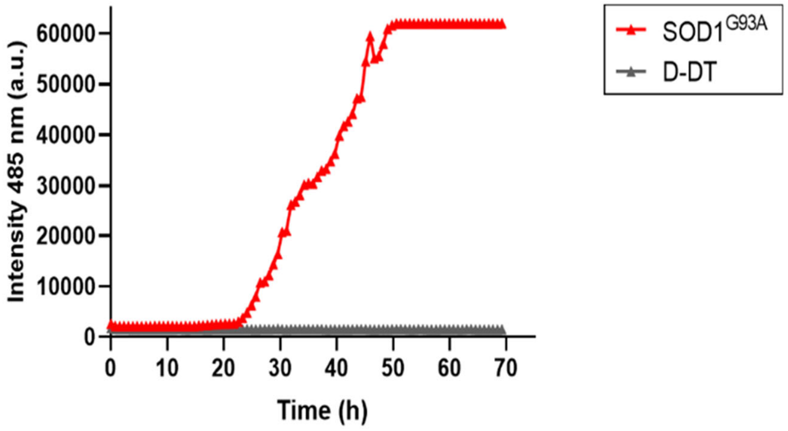

Figure S3: Recombinant D-DT incubated alone does not form amyloid aggregates. ThT fluorescence was monitored for 72 hours during the incubation at 37 °C with continuous shaking of recombinant SOD1G93A alone (50 µM, red) and recombinant D-DT alone (10 µM, gray).

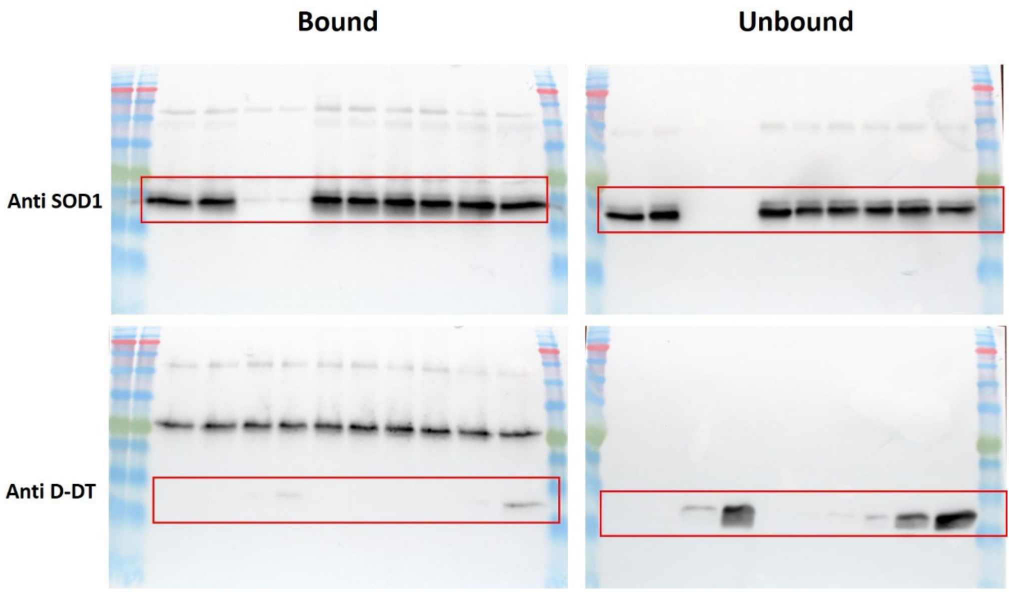

Figure S4: Full-length gels corresponding to Figure 2

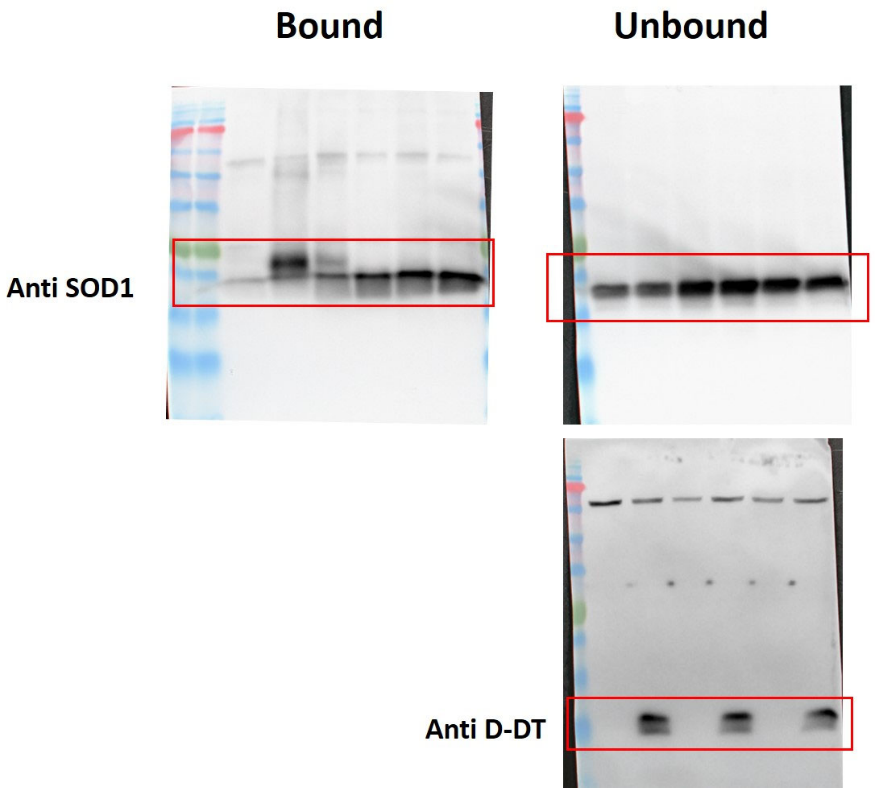

Figure S5: Full-length gels corresponding to Figure 3

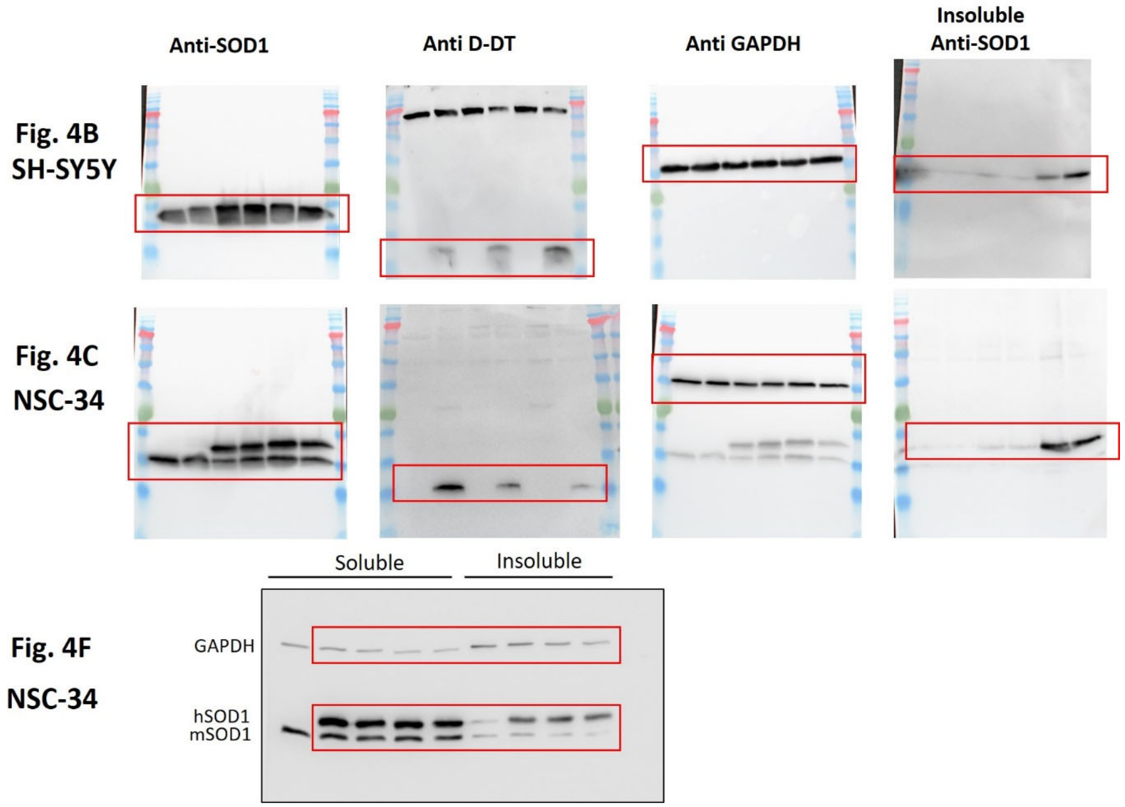

Figure S6: Full-length gels corresponding to Figure 4

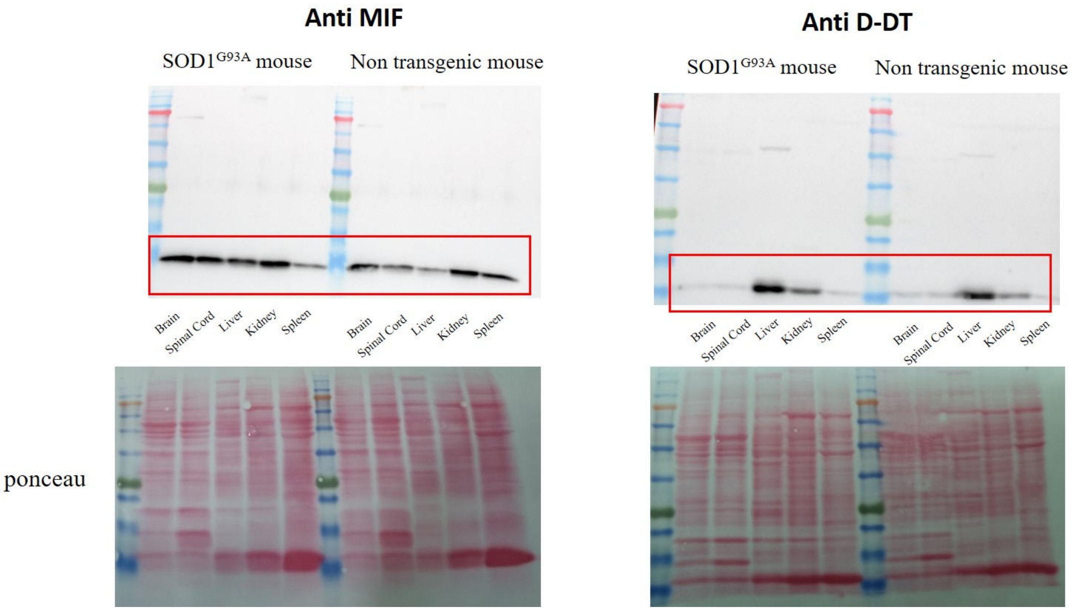

Figure S7: Full-length gels corresponding to Figure 5
